# Fluoroalkenyl-Grafted Chitosan Oligosaccharide Derivative: An Exploration for Control Nematode *Meloidogyne* Incognita

**DOI:** 10.3390/ijms23042080

**Published:** 2022-02-14

**Authors:** Zhaoqian Fan, Yukun Qin, Song Liu, Ronge Xing, Huahua Yu, Kecheng Li, Pengcheng Li

**Affiliations:** 1 CAS and Shandong Province Key Laboratory of Experimental Marine Biology, Center for Ocean Mega-Science, Institute of Oceanology, Chinese Academy of Sciences, Qingdao 266071, China; fzq3707@163.com (Z.F.); ykqin@qdio.ac.cn (Y.Q.); sliu@qdio.ac.cn (S.L.); xingronge@qdio.ac.cn (R.X.); yuhuahua@qdio.ac.cn (H.Y.); lkc@qdio.ac.cn (K.L.); 2 Laboratory for Marine Drugs and Bioproducts, Pilot National Laboratory for Marine Science and Technology (Qingdao), Qingdao 266237, China

**Keywords:** chitosan oligosaccharide, nematicide, *Meloidogyne incognita*, trifluorobutenyl group

## Abstract

The exploration of novel, environmentally friendly, and efficient nematicides is essential, and modifying natural biomacromolecules is one feasible approach. In this study, 6-O-(trifluorobutenyl-oxadiazol)-chitosan oligosaccharide derivative was synthesized and characterized by FTIR, NMR, and TG/DTG. Its bioactivity and action mode against root-knot nematode *M. incognita* were estimated. The results show that the derivative shows high nematicidal activity against J2s, and egg hatching inhibitory activity at 1 mg/mL. The derivative may affect nematode ROS metabolism and further damage intestinal tissue to kill nematode. Meanwhile, by synergism with improving crop resistance, the derivative performed a high control effect on the nematode with low phytotoxicity. These findings suggested that chitosan oligosaccharide derivatives bearing fluoroalkenyl groups are promising green nematicides.

## 1. Introduction

As a kind of soil worm, plant-parasitic nematode (PPN) is one of the global pathogens affecting agricultural production and causing economic losses of up to USD 157 billion annually [1]. Its species are diverse, among which root-knot nematodes (RKNs; i.e., *Meloidogyne* spp.) are the most ubiquitous and notorious [2]. RKN can infect over 2000 plant species [3], especially in fruit and vegetable crop. The control of these pests mainly depends on chemical nematicides. However, nematicide products account for only 2.5% of the global pesticides. The proportion is low compared to the great losses due to PPN [4]. More importantly, many chemical nematicides have been banned or restricted due to safety or environmental problems. Therefore, it is urgent to develop novel and eco-friendly nematicides.

Currently, biocontrol agents based on the biomolecules themselves or their derivatives have received much attention. These agents pose less risk to humans and animals than their synthetic predecessor did, have a selective mode of action, and avoid the emergence of resistant races of pest species [5]. Therefore, they can be applied to integrated pest management (IPM) programs, which tend to use natural or green methods to control crop diseases below the threshold. Chitosan oligosaccharide (COS) is an alternative biological macromolecule worthy of deep research in pest control.

COS, containing 2–20 D-glucosamine units, is produced by the degradation of chitin or chitosan, which is the only cationic compound found in nature. Due to its excellent biocompatibility and unique physiological and biological activity, COS has been widely used as a biostimulant, antifungal agent, seed treatment agent, soil conditioner, and fertilizer in agriculture. As biostimulants, COS can enhance the crop disease resistance by stimulating the secretion of immune enzymes and compounds (such as salicylic acid and jasmonic acid) [6], cell wall reinforcement [7], production of reactive oxygen species [8], and hypersensitive response-mediated cell death [7]. These immune responses are also effective against RKN [9]. Based on this, being endowed with nematicidal activity, COS can act on both nematodes and hosts, thus exerting a desirable control effect in IPM. However, there are few studies on nematicidal COS.

Modification is an effective way to improve the application of natural polymer compounds, including chitin and chitosan [10]. COS can obtain ideal nematicidal activity by grafting active groups. Fluoroalkenyl groups are a kind of active group that are capable of selection. Compounds obtained by combining fluoroalkenyl groups with thiazole, oxazole, or other heterocyclic structures can perform a high nematicidal activity [11,12]. Meanwhile, the fluoroalkenyl compounds also possess lower toxicity to vertebrates than organophosphate- or carbamate-based nematicides [12]. However, these compounds have a risk of phytotoxicity [13], a defect that is expected to be avoidable by combination with COS. In the previous study, we grafted the structure of trifluoroethylene and thiadiazole onto the nitrogen position of COS to obtain a derivative with high nematicidal activity and low phytotoxicity [14], which confirmed the feasibility of fluoroalkenyl COS derivative on nematode control. However, the effect of derivatization based on other microstructures or grafting sites on activity and the action mechanism needs to be studied.

In this paper, one 6-O-(trifluoroebutylene-oxadiazol)-COS derivative was synthesized. Meanwhile, its control effect on *Meloidogyne incognita* was comprehensively evaluated by analyzing the hatching rate of eggs and mortality, and the mechanism of action was explored by analyzing the morphology of second-stage juveniles (J2s) and plant resistance. Finally, the phytotoxicity was estimated in vitro. This study was to offer novel fluoroalkenyl COS derivatives and more data support on nematode control by chitin biomacromolecule modification.

## 2. Results

### 2.1. Characterization

**Scheme 1:**
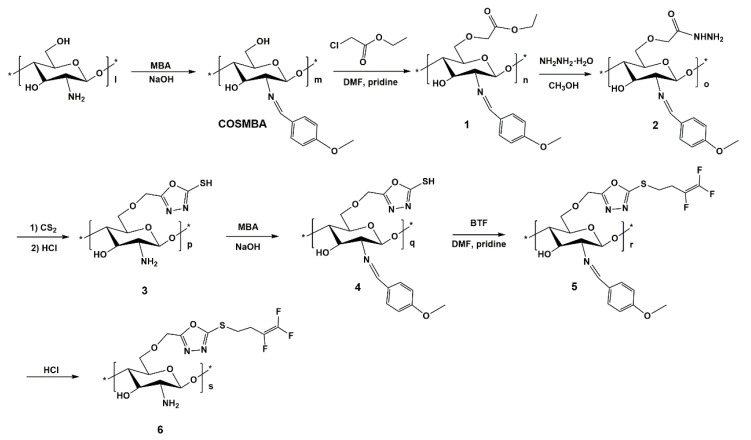
Synthetic route of fluoroalkenyl COS derivative.

In this study, benzaldehyde was selected to protect the amino group of COS, and then a series of 6-substituted derivatives were synthesized according to Scheme 1. The derivatives were charactered by Fourier transform infrared (FTIR), NMR, and thermal gravity analysis and differential thermal gravity (TG/DTG), and the results are shown below.

**Figure 1:**
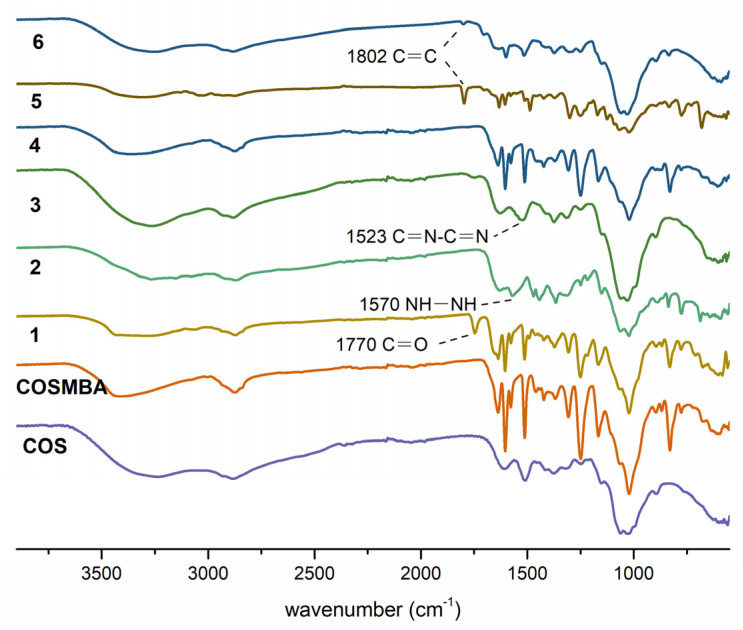
FTIR spectra of fluoroalkenyl COS derivative.

Firstly, the acetoxy structure was introduced into the 6-site (derivative **1**), and the characteristic peaks appeared at 1770 cm^−1^ in the infrared spectrum (Figure 1). When the group reacted with hydrazine, the hydrazide group formed. Thus, the characteristic peak of derivative **2** appeared at 1560 cm^−1^. The synthesis of oxadiazole (derivative **3**) requires acidic conditions. As a result, the amino protection structure was removed, and its 1480 cm^−1^ characteristic peak (C=O absorption peak, benzene ring characteristic peak) disappeared. Concurrently, the signal peak of oxadiazole (1523 cm^−1^) appeared. To ensure that the trifluorobutylene structure was grafted onto the oxadiazole ring, the Schiff base structure was still needed to protect the exposed amino structure in the next step, so the protective group signal appeared and disappeared in compounds **4**–**6** successively. Finally, the characteristic peak of C=C of derivatives **5** and **6** appeared at 1802 cm^−1^, indicating that the trifluorobutylene group had been introduced.

**Figure 2:**
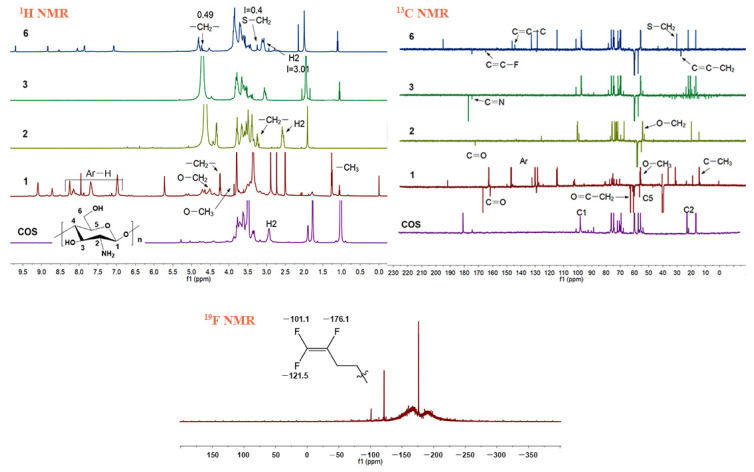
^1^H NMR, ^19^F NMR, and ^13^C NMR spectra of fluoroalkenyl COS derivative.

When the Schiff base and acetoxyethyl groups were introduced into the COS skeleton, the complex signal changes appeared in the ^1^H NMR (Figure 2). Among them, δ 7.0–8.5 ppm is Ar–H signal peak. For acetoxyethyl, the –CH_3_ peak is in a high field (δ 1.24 ppm), and two methylene groups are O=C–CH_2_ at δ 4.23 ppm and O–CH_2_ at δ 3.80 ppm. The former did not disappear with deprotection or cyclization. However, it showed different shifts with different connecting groups, such as δ 3.34 ppm of derivative **2**, δ 4.69 ppm of derivative **3,** and δ 4.79 ppm of derivative **6**. There are impurity peaks in the spectrum of derivative **2**, but they are removed in the next step. For derivatives **3** and **6**, there is no new group containing hydrogen bonds, so the hydrogen spectrum changes little. The derivative **6** contains trifluorobutylene structure, and its S–CH_2_ is in the range of δ 3.25 ppm, C=C–CH_2_ at δ 3.05 ppm, which overlaps with the H2 peak of COS. Additionally, according to the calculation of H2 and S–CH_2_ peak integrals, the degree of substitution of trifluorobutylene group was 6.6%.

The ^13^C NMR is shown in Figure 2. The carbonyl peak of acetoxyethyl of derivative **1** appears in the δ 167.2 ppm and it then shifted to δ 172.4 ppm when the hydrazine group was added. After cyclization (derivative **2**), the carbonyl peak disappears and the C=N peak appears at δ 174.6 ppm. The ethylene bond peaks of the trifluorobutylene group appear at δ 144.5 ppm and δ 165.5 ppm, and the two methylene peaks were at δ 27.2 ppm and δ 30.3 ppm. Combined with the shifts of three fluorine atoms of derivative **6** at δ 101.1 ppm, 125.1 ppm, and δ 176.1 ppm in ^19^F NMR (Figure 2), it can be ascertained that the target compound has been successfully synthesized.

**Figure 3:**
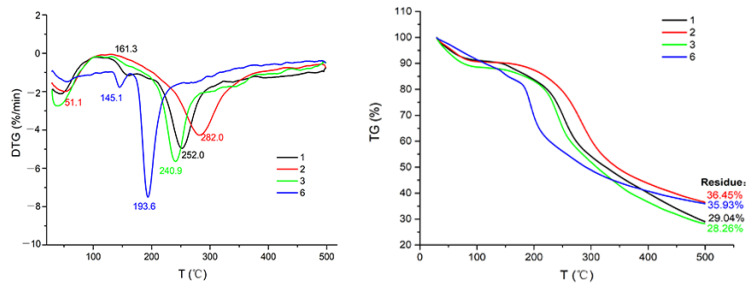
DTG and TG thermogram of fluoroalkenyl COS derivative.

Thermal gravity thermogram can exhibit the thermal stability of derivatives. As shown in Figure 3, there are two decomposition peak temperatures (T*_d_*) in derivative **1**, the former is smaller (161.3 °C), which is due to the corrosion of the Schiff base structure, and the latter (252.0 °C) is attributed to the degradation of the main structure. When the hydrazide group formed, the stability was improved. Therefore, derivative **2** had a higher T*_d_* (282.0 °C) than that of derivative **1**. For derivative **3**, the structure of oxadiazole is stable, and the T*_d_* is close to 250 °C. However, the stability of the trifluoro butenyl oxadiazole sulfide structure is poor, so it corroded rapidly in a relatively low temperature (193.6 °C), and then the depolymerization and degradation of the COS skeleton are relatively gentle. In addition, the small peat at 145 °C may be attributed to the residual Schiff base structure. Nevertheless, the above COS derivatives are stable at room temperature, which is conducive to activity evaluation. However, derivatives **4** and **5** were water-insoluble, hence their activity and thermal stability were not estimated here.

### 2.2. Effect of Derivative on Egg Hatching

**Figure 4:**
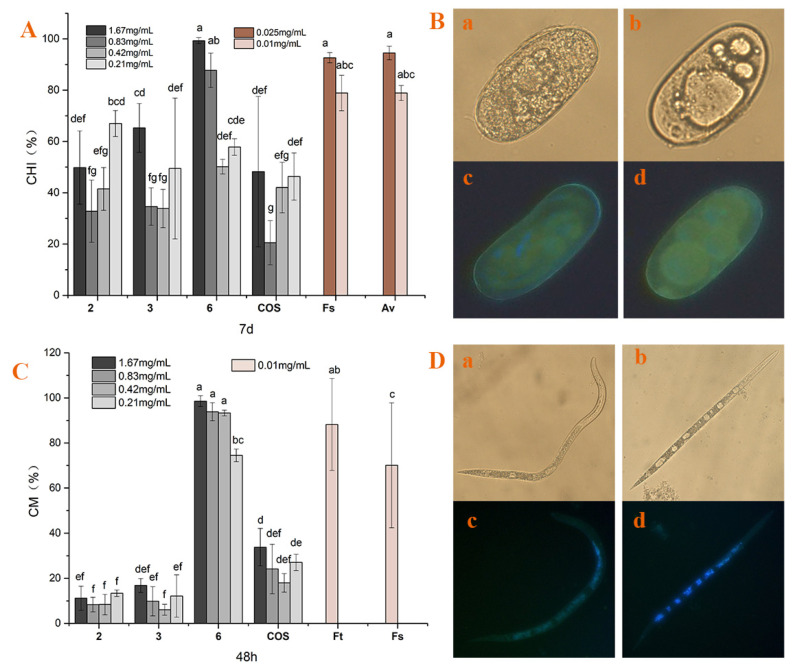
Effects of COS derivatives on eggs and J2s: (**A**) was egg hatching inhibitory activity at 7 d; (**C**) was nematicidal activity at 48 h; (**B**) or (**D**) was the morphology of egg and J2 under stereomicroscope and fluorescence microscope, of which (**a**,**c**) belong to the blank control, (**b**,**d**) were treated with 1 mg/mL of derivative **6**. Fs, Ft, and Av represent positive control fluensulfone, fosthiazate, and avermectin, respectively. Different letters (a, b, c, etc.) above histogram mean statistical significance at the *p* ≤ 0.05 level.

Root-knot nematode develops into first instar larval (J1) in the egg, breaks shell at J2 stage, and quickly infects the root of the surrounding plant. Inhibiting egg hatching can reduce the incidence of nematode diseases. In this study, derivatives **2** and **3** have no difference in the egg hatching inhibition activity with COS (Figure 4A), while derivative **6** has higher inhibition activity. At 0.83 mg/mL, its egg hatching inhibition rate (CHI) can reach more than 80% and even 100% at higher concentrations. It indicates that alone the hydrazide and oxadiazole structure has little influence on the activity. Conversely, the combination of oxadiazole and trifluorobutylene can significantly improve the activity. Derivative **6** can cause some eggs to produce vacuoles (Figure 4B). There is no special fluorescence in the vacuole, which is speculated to be lipid particles. Although it is hard to explain how the active substance penetrates the eggshell, it is certain that the derivative causes toxicity to the egg cells and blocks the hatching process.

### 2.3. Toxicity of Derivative to J2s

Controlling the number of J2, the stage of host-seeking and infecting is the first aim of all kinds of nematicides. Compared with other derivatives, derivative **6** displayed higher nematicidal activity against J2s (Figure 4C). The corrected J2s mortality (CM) of 48 h can reach 80% at 0.42 mg/mL, and the value can reach 100% with the increase in time, which exceeds the effect of Fs at 0.01 mg/mL. The result showed that the combination of oxadiazole and trifluorobutylene on the nitrogen position of chitosan has high toxicity to J2s. It is similar to the combined action of thiazole and trifluorobutylene on nitrogen position in previous research [14]. These data indicated that thiazole, oxazole, or other nitrogen-containing heterocyclic structures have a strong synergistic effect with the trifluorobutylene group, thereby improving the nematicidal activity of COS at any grafting site.

**Figure 5:**
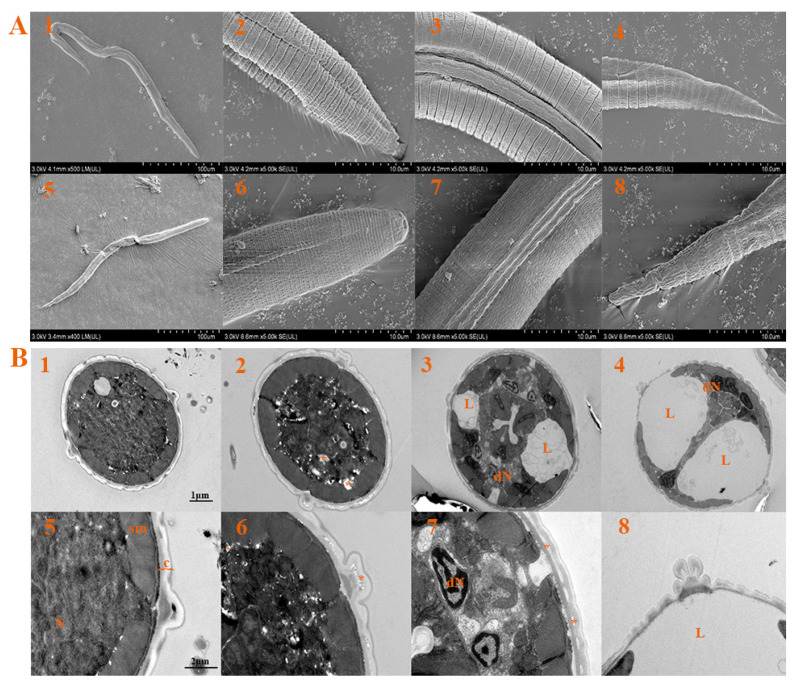
Scanning electron microscopy (**A**) and transmission electron microscopy (**B**) micrographs of the cuticle of *Meloidogyne incognita* juveniles: A1–A4, B1, and B5 belong to the blank control group; others were treated with derivative **6**. Note: c = cuticle, sm = somatic muscles, N = nucleus, * = electron-lucent cavity, L = lipid granule, dN = nucleus depolymerization.

In order to investigate how derivative **6** causes J2 death, we observed its effect on the surface and internal tissues by scanning electron microscopy (SEM) and transmission electron microscopy (TEM). The body surface of J2 is covered with annuli that are separated by furrows. Meanwhile, the wrinkled lateral cord and alae are distributed on both sides of the body (Figure 5A). After being treated with derivative **6**, the furrows in the head and middle become narrower, and the alae become smooth. In addition, the cuticle was not significantly damaged.

The cuticle of *M. incognita* J2 was built with exocuticle, intermediate zone, and lamellar endocuticle (Figure 5B). The underlying layer was somatic muscle (sm) with clearly seen myosin fibers [15]. The center was pseudocoel filling with homogenous syncytial gut cells, and the nuclei were electron-dense and had no obvious abnormality. However, after treatment with derivative **6**, several small electron-lucent cavities (*) were visible in the coelom and the nucleus depolymerized. Meanwhile, electron-lucent areas also appeared in the intermediate zone of the cuticle and expanded into vacuolization. Derivative **6** caused nuclear condensation in the coelom and lipid granules fusion into large macroscopic vacuoles. Unlike the continuous globular lipid droplets that often appear in the death of nematodes caused by other nematicidal compounds [16,17], the lipid vacuoles were plump and separated by cytoplasm, and were particularly obvious under the intestinal autofluorescence, which may be induced by lipofuscin (Figure 4D).

### 2.4. Control Effect of Derivatives

**Figure 6:**
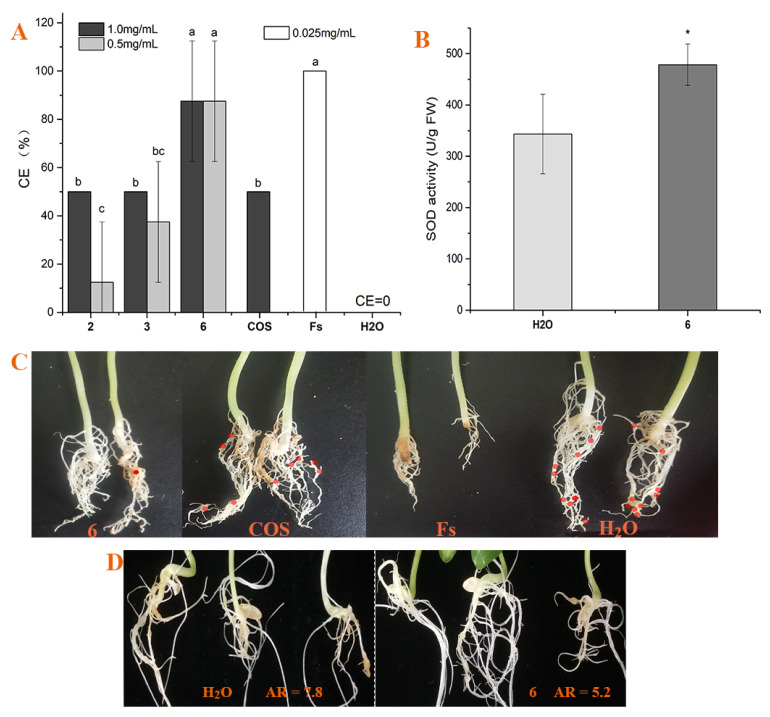
Control effect of COS derivatives on *Meloidogyne incognita*: (**A**) was the control effect of COS derivatives; (**B**,**C**) were the effect of derivative **6** on root SOD, where the red point represents the root-knot; (**D**) induced resistance activity of the derivative, among which AR was the average number of root-knot. Different letters (a, b, and c, etc.) and * above histogram means different at *p* ≤ 0.05 according to *t*-test.

As shown in Figure 6A,B the control effect of derivatives **2** and **3** was similar to that of COS, which was consistent with the results of in vitro assay and was not high. In contrast, the root-knot number of the treatment group of derivative **6** was small, and the control effect was up to 87.5%. Although its control effect was slightly weaker than that of the positive control, it did not inhibit the root growth of cucumber seedlings. This suggests that the derivative **6** has a positive effect on practical control. Therefore, we also investigated its induce resistance.

As shown in Figure 6C, the root-knots distributed on the taproot in series and the fibrous root near the hypocotyl individually, in a state of inflating and string. The newly grown roots had no root-knots distribution, indicating that nematodes invade radicles in the early stage of the experiment and became dispersed with root development. At this stage, fewer nematodes invaded the roots of the treatment group than that of the blank control group, forming the less average numbers of root-knots (5.2 < 7.8). Additionally, derivative **6** enhanced superoxide dismutase (SOD) content in cucumber roots (Figure 6D), which is an important immune enzyme participating in the redox regulating cascade. Therefore, derivative **6** can improve the resistance of cucumber against *M. incognita* and enhance the control effect, meaning the 6-O-grafted fluoroalkenyl structure could not shield the inducing activity of the chitosan skeleton on the crop.

### 2.5. Phytotoxicity

**Figure 7:**
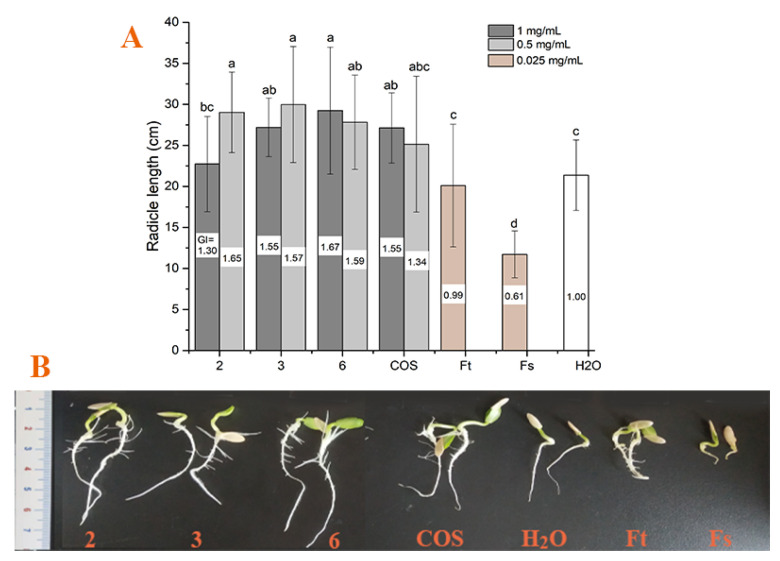
Phytotoxicity of COS derivatives: (**A**) was the effect on radicle length, and the number in the center of histogram is GI value; (**B**) was the effect on seed germination. Different letters (a, b, c, etc.) above histogram mean statistical significance at the *p* ≤ 0.05 level.

As shown in Figure 7, at 0.5 mg/mL, the radicle length of all sample treatment groups was higher than that of the blank group. At the same time, after the sample treatment, the radicle of the seeds grew further, and the fibrous roots were significantly more than those of the blank. All derivatives can promote the growth of radicle, and the germinating finger (GI) values were significantly greater than 1.00. On the contrary, fluensulfone inhibited the radicle growth to a certain extent with GI < 1.00, while fosthiazate had little difference to the blank group, but the growth was weak. These results indicate that the derivatives avoid the defect of fluoroalkenyl groups and have low phytotoxicity.

## 3. Discussion

Plant, nematode, and soil environment interact to form a complete ecosystem. Strengthening infection barriers, reducing the number of living bodies, and changing survival environments are efficient ways to control nematode diseases. The combination of the three would play a more efficient nature control function, which conforms to the strategy of IPM. COS derivative may control nematode from the above three pathways at the same time. Chitin or chitosan can reduce the nematode reproduction factor and its population in the soil by mediated chitinolytic microorganisms [18,19,20]. COS and its derivatives may possess similar efficiency. However, as the soil microenvironment is complex, it will not be discussed here.

Grafting exogenous active groups can undoubtedly improve the nematicidal activity of COS, however, there is no report on how derivatives affect the physiology of nematodes. In this study, derivative **6** did not significantly influence the cuticle, therefore, it was likely to be swallowed by nematodes into the intestine to work. The intestinal autofluorescence in nematode is caused by lipofuscin, a marker of aging and oxidative stress induced by reactive oxygen species (ROS) [21]. Exposure to toxins can increase ROS levels, leading to lipid peroxidation, which eventually produces lipofuscin. The lipofuscin will deposit on the cells and accelerate aging [22]. In this study, autofluorescence of nematode was significantly enhanced after being treated (Figure 4D), indicating that the derivative **6** may lead to lipofuscin accumulation and accelerate aging by affecting ROS metabolism. ROS also change the architecture of cell membranes and damage tissue and cellular components [16]. In this study, ROS may be involved in the intestinal cell damage accompanied by aging, including nuclear depolymerization, cell shrink and rupture, and lipid vacuoles forming et ac.

The inducible activity of derivatives may attribute to the COS skeleton. As an elicitor of plant innate immunity, COS can trigger plant responses against fungal and bacterial infection, including Ca^2+^ spiking, NO and ROS accumulation, activation of the MAPK cascade, upregulation of defense gene expression, activation of the SA and JA-mediated signaling pathways, callose deposition, and molecular flux via plasmodesmata [23,24]. Although nematode and fungi or bacteria are completely different organisms in both lifestyle and infection modes, the immune responses of plants to the two diseases are similar. For example, ROS plays a major role in plant-nematode interactions. ROS stimulates genes of the phenylpropanoid pathway and enhances the synthesis of phenols by L-Phenylalanine ammonla-lyase [8], resulting in formatting lignin and suberin that strengthen the physical barrier of the cell wall [25]. ROS accumulation can lead to defense-related cell death or a hypersensitive response, which helps to isolate the pest [26]. ROS also amplify and propagate intra- and intercellular defense signals, activating the long-lasting resistance in non-infected tissues as well (termed systemic acquired resistance) [27]. Besides, ROS largely relies on the accumulation of salicylic acid (SA) at the site of infection [27]. SA or jasmonic acid, which mediate systemic acquired resistance, lead to the induction of pathogenesis-related proteins, which are important signaling molecules in plant immunity [28]. Boosting the SA pathway, which was stimulated by the pathogen or exogenous inducer, might play a defensive role against newly hatched J2s during the early stages of nematode invasion [29]. In this study, the target derivative improved SOD activity, an antioxidant enzyme with an elevation in the level of ROS. Therefore, the above immune substance may be involved in the resistance of cucumber to *M. incognita* improved by derivative **6**.

Usually, prevention is the main control method of root-knot nematodes, because once nematode larvae invade plant tissues, ordinary nematicides will find it difficult to produce a marked control effect as an in vitro assay. Therefore, the derivative with nematicidal activity and inducing activity will have a greater advantage in dealing with crops in the disease stage. Enhancing resistance and improving growth will weaken the damage caused by nematode invasion and slow down the treatment threshold in IPM. In addition, compared with fluoroalkenyl compounds, the derivative avoided the risk of phytotoxicity and will have safer application methods.

## 4. Experimental

### 4.1. Materials and Methods

Chitosan oligosaccharide (Mw < 3000 Da, DD > 85%) was purchased from Shanghai Yuanye Biotechnology Co., Ltd. (Shanghai, China). The 4-Bromo-1,1,2-trifluoro-1-butene (98%) (BTF) was purchased from Aladdin Reagent (Shanghai, China) Co., Ltd. Hydrazine hydrate (80%), Hydrochloric acid, and other analytical reagents were purchased from Sinopharm Chemical Reagent Co., Ltd. (Beijing, China). SOD activity kit was purchased from Nanjing Jiancheng Bioengineering Institute (Nanjing, China).

FTIR spectra were performed ranging from 4000 cm^−1^ to 400 cm^−1^ using a Thermo Scientific Nicolet iS10 FT-IR spectrometer (Waltham, MA, USA). The ^1^H NMR, ^13^C NMR, and ^19^F NMR were recorded with a JEOL JNM-ECP600 spectrometer (Tokyo, Japan), using D_2_O as solvents. A METTLER TGA-DSC 1 SF/1382 (Zurich, Swiss Confederation) was used to record the TG/DTG curves of the polymers from 25 to 500 °C in nitrogen, and the heating rate was 10 °C/min.

### 4.2. Synthesis of COS Derivatives

Synthesis of N-(4-methoxybenzylidene) chitosan oligosaccharide derivative (**COSMBA**).

In order to ensure the water solubility of the target derivative, the amino group of COS needs to be protected with Schiff base (Scheme 1). Briefly, COS (10 g) and sodium hydroxide (4 g) were dissolved in distilled water (100 mL), then p-methoxybenzaldehyde (9.1 mL) was added to react for 10 h at room temperature. After filtration, the filter cake was washed with distilled water and ethanol successively and dried to obtain COSMBA. The yield was 82.5%.

Synthesis of 6-(2-ethoxy-2-oxoethoxy)-N-(4-methoxybenzylidene) chitosan oligosaccharide derivative (**1**).

For product derivative **1**, COSMBA (6 g) and pyridine (4 mL) were mixed in N, N-Dimethylformamide (DMF) (40 mL), then ethyl chloroacetate/DMF solution (6.75/10 mL) was dripped in an ice bath. After reaction for 12 h at room temperature, acetone was added to the mixture to precipitate. The sediment was washed with absolute ethanol and dried at 60 °C to obtain a tawny solid. The yield was 62.5%.

Synthesis of 6-(2-hydrazinyl-2-oxoethoxy)-N-(4-methoxybenzylidene) chitosan oligosaccharide derivative (**2**).

To produce derivative **2**, derivative **1** (5.9 g) and hydrazine hydrate (6 mL) were added in methanol (50 mL) and refluxed for 10 h. After filtration, the filter cake was washed with absolute ethanol and dried at 60 °C, and a yellow-white solid was obtained. The yield was 47.1%.

Synthesis of 6-((5-mercapto-1,3,4-oxadiazol-2-yl)methoxy) chitosan oligosaccharide derivative (**3**).

A mixture of derivative **2** (2 g), sodium hydroxide (0.6 g), and carbon disulfide (1.6 mL) in ethanol (20 mL) was heated to 60 °C for 7 h. After the reaction, the ethanol was removed by vacuum distillation. The residue was dissolved in water, adjusted to pH 3–4 with 3% hydrochloric acid, and stirred for 1 h. Derivative **3** was obtained by concentrating, adding ethanol to precipitate, filtering, washing filter cake with anhydrous ethanol, and drying at 60 °C. The yield was 85.0%.

Synthesis of 6-((5-mercapto-1,3,4-oxadiazol-2-yl) methoxy)-N-(4-methoxybenzylidene) chitosan oligosaccharide derivative (**4**).

The synthesis of derivative **4** was similar to COSMBA. Briefly, derivative **3** (1.6 g) and sodium hydroxide (0.8 g) were dissolved in distilled water (20 mL), then p-methoxybenzaldehyde (1.45 mL) was added to react for 10 h at room temperature. After filtration, the filter cake was washed with distilled water and ethanol successively and dried to obtain the Schiff base derivative. The yield was 93.1%.

Synthesis of 6- ((5-((3,4,4-trifluorobut-3-en-1-yl) thio)-1,3,4-oxadiazol-2-yl)methoxy) -N-(4-methoxybenzylidene) chitosan oligosaccharide derivative (**5**).

Derivative **4** (1.3 g), pyridine (2 mL), and BTF (1.12 mL) with 1.1 times molar mass were added in DMF (20 mL) successively and raised to 70 °C for reaction overnight. After filtering at room temperature, the filter cake was washed with anhydrous ethanol and dried to obtain a yellowish-brown solid. The yield was 31.7%.

Synthesis of 6- ((5-((3,4,4-trifluorobut-3-en-1-yl) thio)-1,3,4-oxadiazol-2-yl)methoxy) chitosan oligosaccharide derivative (**6**).

A mixture of derivative **5** (0.4 g) with acetone (10 mL) was adjusted to have a pH of 3–4 by diluted hydrochloric acid (3 M) and stirred for 1 h. Finally, the precipitate was collected, washed with ethanol, and dried to obtain the target derivative. The yield was 60.6%.

### 4.3. Nematicidal Assay In Vitro

#### 4.3.1. Nematode Populations Collection

*M. incognita* eggs were collected from infected tomato roots cultured in the laboratory. Briefly, the roots were cut into 1 cm-long pieces and shaken in 0.6% (*w*/*v*) sodium hypochlorite solution for 4 min. The segments were successively sieved by 200 mesh and 500 mesh screens, then washed with plenty of water. The eggs were collected with sterile water and the suspension was prepared (2500 eggs/mL). For obtaining J2s, the eggs were put into Baermann funnels to hatch for 3–5 days at 28 °C. J2s collected at 1–2 days were eliminated. The eggs and J2s were observed with a stereo-microscope, optical microscope, and fluorescence microscope (excitation: 488 nm, emission: 510 nm) as required.

#### 4.3.2. Egg Hatching Assay

Egg suspension (40 μL) was placed into a 48-well culture plate, and the number of eggs was counted. Then, 200 μL of derivative solution (2, 1, 0.5, 0.25, 0.125 mg/mL) was added into each well. The plates were placed in the dark environment at 25 °C and J2 numbers were counted at 7th day. Sterile water was used as blank control, fluensulfone and avermectin (Av) in 1% Tween20 solution (0.01 and 0.025 mg/mL) were used as positive controls, and each experiment was repeated three times. The hatching inhibition activity is expressed by the corrected hatching inhibition rate (CHI):

$$hatching\;rate\;of\;eggs\;\left(HR\right)\;\left(\%\right)=\frac{J2\;numbers}{egg\;numbers}\times 100$$

$$CHI\;\%=\frac{HR\;of\;blank\;control-HR\;of\;treatment}{HR\;of\;blank\;control}\times 100$$

#### 4.3.3. Nematicidal Activity

The evaluated method of nematicidal activity was similar to the egg hatching assay. Briefly, 40 μL of J2s suspension and 200 μL of derivative solution (2, 1, 0.5, 0.25, 0.125 mg/mL) were added into 48-well culture plate. After being cultured for 48 h, the number of dead J2s (rigidity nematode) was counted. In this experiment, fosthiazate (Ft) and fluensulfone were used as positive controls, and each experiment was repeated three times. The nematicidal activity was evaluated by CM, which was calculated as the followed equation:

$$J2s\;mortality\;\left(JM\right)\;\left(\%\right)=\frac{number\;of\;dead\;J2s}{total\;number\;of\;J2s}\times 100$$

$$CM\;\left(\%\right)=\frac{JM\;of\;treatment-JM\;of\;blank\;control\;}{1-JM\;of\;blank\;control}\times 100$$

### 4.4. In Vivo Control Effect Assay

The control effect of COS derivative on *M. incognita* was estimated by the greenhouse test tube method. Briefly, the river sand was sifted by a 20-mesh sieve, sterilized, then placed in a glass tube (2 × 10 cm). Next, cucumber seeds that had had their germination accelerated were dibbled and then placed in a light incubator. The culture condition was 12 h, 26 °C, and light intensity 15,000 Lux in the daytime; 12 h, 22 °C at night; the humidity was 70–80%. After seven days, 0.5 mL of sample solution (1.0 and 0.5 mg/mL) and fluensulfone solution (0.025 mg/mL) were applied. On the next day, 100 eggs were inoculated. There were left to continue to culture for 15 days, and the root knots were counted in water for disease classification (DC). In this assay, the water was blank control, and every experiment was replicated three times. The DC standard was described as follows:

Level 0: no visible root knot. Level 1, level 2, level 3, and level 4 represent root caking rates of 1–25%, 26–50%, 51–75%, and 76–100%, respectively.

The effect of derivative on root-knot nematode disease was estimated by control effect rate (CE), which was calculated as the following formula:

$$\mathrm{Disease}\;\mathrm{index}\;\left(\mathrm{DI}\right)=\frac{\sum \;\left(DC\;value\times the\;number\;of\;disease\;plants\;on\;relative\;DC\right)}{total\;number\;of\;investigated\;plants\times maximal\;DC\;value}$$

$$CE\;\left(\%\right)=\frac{DI\;of\;blank\;control-DI\;of\;treatment}{DI\;of\;blank\;control}\times 100$$

### 4.5. Scanning Electron Microscopy and Transmission Electron Microscopy

For SEM, 1000 J2s were immersed in 1 mg/mL of derivative **6** solutions overnight, then collected by centrifugation (5000 rpm/min, 4 min). After washing with water three times, the J2s were fixed with 2.5% glutaraldehyde at 4 °C overnight. Next, the J2s were washed with 0.1 M of pH 7.0 phosphate buffer, dehydrated in an ethanol gradient (30–100% ethanol; *v*/*v*), and sprayed with gold ion sputter for SEM using a Hitachi SU8020 field-emission scanning electron microscope (Hitachi, Japan).

For TEM, J2s were fixed with 2.5% glutaraldehyde overnight and 1% osmic acid for 1–2 h, respectively. Then, the specimens were dehydrated in an ethanol gradient (30–100% ethanol; *v*/*v*) for 15 min and acetone for 20 min, and treated with different ratios of resin/acetone solution (1:1 for 1 h, 3:1 for 3 h, and 1:0 at 70 °C overnight; *v*/*v*). The embedded materials were cut in Leica EM UC7 Ultramicrotome to obtain 70–90 nm slices. The slices were stained with uranyl acetate and lead citrate staining solution for 5 min, respectively, and then imaged with a JEM-1200ex transmission electron microscope (JEOL, Japan).

### 4.6. Induced Resistance Assay

The germinating cucumber seeds were soaked in 0.2 mg/mL of derivative **6** solutions and distilled water until the radicle grew to 1 cm long (about one night). Then, two seeds of two groups were washed with water and placed symmetrically on the edge of a φ9 cm petri dish covered with 5 mL of Pluronic F-127 gel (23%) at room temperature, respectively. The gel was prepared according to the method described by Zhan et al. [30]. Next, 100 J2s were placed in the center of the dish and incubated in a dark environment for 48 h. The seedlings of the two groups were washed with distilled water and cultured in a humid environment for 7 days. Finally, the average number of root-knots (AR) was counted, and every experiment was replicated three times.

### 4.7. Root SOD Activity Assay

As above assay, cucumber seeds were cultured in a humid environment until roots grew to 4 cm long. A total of eight seedlings were selected and treated with 1 mg/mL of derivative **6** or water (blank control) for 48 h. Then, every two roots were washed with distilled water, ground into pulp with quartz sands, and diluted to 10 mL with phosphoric acid buffer (50 mM, pH 7.8). After centrifugation at 8000 rpm for 10 min at 4 °C, the supernatant was collected to detect SOD activity according to kit instructions. The SOD content was represented as U/g fresh root.

### 4.8. Phytotoxicity Assay

In this assay, 10 uniform and plump cucumber seeds were chosen and put in a φ9 cm petri dish lined with filter paper. Then, 4 mL of derivative solution (0.5 and 1.0 mg/mL) and positive control solution (0.025 mg/mL) were added. The seeds were cultured in the dark at 25.0 °C. After 48 h, the germination rate and radicle elongation were determined. Each treatment was repeated three times, and distilled water was used as blank control. GI was calculated by the following formula:

GI=(RLs×GSs)/(RLc×GSc)

where the letter s and c represent the results of the sample treatment group and the blank control group, respectively; RL and GS denote the length of radicle and the number of germinating seeds, respectively. When GI exceeds 1, the derivative is considered to have no phytotoxicity; conversely, GI lower than 0.8 is considered to be phytotoxic.

### 4.9. Data Analysis

Statistics were performed using SPSS 24 statistical software. Except for data of SOD activity analyzed using Tukey’s HSD test, all data were determined using Duncan’s multiple range test. In all histograms, different letters (a, b, c, etc.) or symbols (*) were used to represent statistical significance at the *p* ≤ 0.05 level.

## 5. Conclusions

The present study results show that trifluorobutenyl with oxadiazol groups grafting on 6-O position can improve the bioactivity of COS against root-knot nematode *M. incognita*. Through reducing the number of J2s and enhancing plant resistance, derivative **6** performs a high control effect on *M. incognita*. As a consequence, the fluoroalkenyl COS derivative possesses alternative modes of action and environmental sustainability, which offer the possibility for use in integrated pest management. This discovery provides a new template for green nematicide exploration-based biomacromolecules. However, whether COS derivatives can exert more natural factors to control nematodes in IPM needs to be studied further; future studies could examine the influence on soil microorganisms and the practical control effect in soil.

## Abbreviations

AR, average number of root-knots; Av, avermectin; BTF, 4-Bromo-1,1,2-trifluoro-1-butene; CE, control effect rate; CHI, hatching inhibition rate; CM, corrected J2s mortality; COS, chitosan oligosaccharide; DC, disease classification; DMF, N,N-Dimethylformamide; Fs, fluensulfone; Ft, fosthiazate; GI, germinating finger; IPM, integrated pest management; J2s, second-stage juveniles; PPN, plant-parasitic nematode; RKN, root-knot nematodes; ROS, reactive oxygen species; SA, salicylic acid; SEM, scanning electron microscopy; SOD, superoxide dismutase; TEM, transmission electron microscopy; TG/DTG, thermal gravity analysis/ differential thermal gravity.
